# An Intelligent Multi-View Active Learning Method Based on a Double-Branch Network

**DOI:** 10.3390/e22080901

**Published:** 2020-08-17

**Authors:** Fucong Liu, Tongzhou Zhang, Caixia Zheng, Yuanyuan Cheng, Xiaoli Liu, Miao Qi, Jun Kong, Jianzhong Wang

**Affiliations:** 1 College of Information Sciences and Technology, Northeast Normal University, Changchun 130117, China; liufc001@nenu.edu.cn (F.L.); zhangtz142@nenu.edu.cn (T.Z.); zhengcx789@nenu.edu.cn (C.Z.); chengyy897@nenu.edu.cn (Y.C.); qim801@nenu.edu.cn (M.Q.); 2 Institute for Intelligent Elderlycare, College of Humanities and Sciences, Northeast Normal University, Changchun 130117, China; 3 Department of Chemical & Biomolecular Engineering, National University of Singapore, Singapore 117585, Singapore; chelxi@nus.edu.sg

**Keywords:** active learning, deep learning, image classification, data analysis and selection

## Abstract

Artificial intelligence is one of the most popular topics in computer science. Convolutional neural network (CNN), which is an important artificial intelligence deep learning model, has been widely used in many fields. However, training a CNN requires a large amount of labeled data to achieve a good performance but labeling data is a time-consuming and laborious work. Since active learning can effectively reduce the labeling effort, we propose a new intelligent active learning method for deep learning, which is called multi-view active learning based on double-branch network (MALDB). Different from most existing active learning methods, our proposed MALDB first integrates two Bayesian convolutional neural networks (BCNNs) with different structures as two branches of a classifier to learn the effective features for each sample. Then, MALDB performs data analysis on unlabeled dataset and queries the useful unlabeled samples based on different characteristics of two branches to iteratively expand the training dataset and improve the performance of classifier. Finally, MALDB combines multiple level information from multiple hidden layers of BCNNs to further improve the stability of sample selection. The experiments are conducted on five extensively used datasets, Fashion-MNIST, Cifar-10, SVHN, Scene-15 and UIUC-Sports, the experimental results demonstrate the validity of our proposed MALDB.

## 1. Introduction

In recent years, computer technologies such as artificial intelligence [1,2] have changed our life a lot. With the significant improvement of computing power, deep convolutional neural networks (CNNs) have become a hot issue in the field of artificial intelligence [3]. Although CNNs have achieved great success in many complex tasks, such as natural language processing, action recognition, network traffic analysis [4,5], mobile encrypted traffic classification [6,7,8], object detection [9] and hyperspectral image analysis [10], they still suffer from a big flaw: training an effective deep CNN model requires a huge amount of labeled data. However, in many real-world scenarios, such labeled data is very scarce. Especially in particular areas such as image and video processing, the amount of available labeled data is even smaller since the tedious labeling process often requires a lot of time and manual labor [11]. To reduce the labeling workload, active learning has been proposed and can achieve good performance when combined with traditional classifiers, e.g., support vector machine (SVM), K-nearest neighbor (KNN) and dictionary learning [12,13]. Recently, active learning has also been introduced into the convolutional neural networks field to alleviate the effort of labeling intelligently, which has resulted in a great performance improvement [14].

Active learning is an iterative progress to choose the most valuable and useful unlabeled data to label for expanding the training dataset [15], which can optimize the learning results as much as possible. In each active learning iteration, the parameters of the model are fine-tuned by the selected valuable samples. The sample selection strategies are the key to active learning, which is heavily dependent on the previous features learned from the current model. The strategies also affect the analysis and evaluation of the unlabeled data by the current model. Therefore, how to design an effective method to choose useful samples from the unlabeled data pool is crucial. The quality of the selection strategy determines whether the selected dataset can effectively contain rich information, remove noise data, and represent the whole dataset [16]. Numerous algorithms have been proposed to find a small informative sample subset so that the model trained on this small subset is comparable to that trained over the whole dataset. According to the different principles of sample acquisition methods, the current active learning techniques are mainly divided into three categories: pool-based, stream-based and learning by query synthesis [17]. Pool-based active learning methods first put all samples in an unlabeled data pool, and then select suitable samples from this pool for labeling. Under this setting, all samples will be provided to the learning model, and the model will select a part of the samples based on some predefined criteria to query their label. In stream-based active learning methods, samples are not stored in the pool, but in a certain order (in the form of data stream) for the model to determine whether or not each newly seen data need to be manually labeled. Query synthesis means the active learning model can generate some artificial samples to reveal sensitive information and improve its learning ability. In recent years, pool-based and stream-based methods become two popular strategies for active learning. Most of these methods choose one of the two criteria [16], i.e., representativeness and informativeness, for data analysis and sample selection. Representativeness and informativeness are designed based on the data distribution and the output of classifier, respectively. The purpose of data distribution-based approach is to build a subset to represent the true distribution of the entire dataset as well as possible [18], while the methods based on the outputs of classifier is much simple and lower in computing complexity. Hence, many active learning methods were proposed by adopting the informativeness as sample selection criterion. However, most of the existing approaches are proposed based on a single classifier rather than the fusion of multiple classifiers. Therefore, if the single classifier is not very effective (include not stable) or has a strong inductive bias, it can hardly characterize the usefulness of the samples well, which will limit the performance and stability of the active learning [19].

Since Wang and Shang [14] applied active learning to deep learning, the strategy of uncertainty sampling is widely used in various deep learning models to estimate the informativeness of samples. However, some studies have pointed out that the samples selected by the uncertainty evaluated only based on the final output in deep learning model are insufficient [20,21]. This is due to the fact the last layer of a deep learning model is task oriented, which ignores the information learned by the middle hidden layers during the data analysis and selection progress. At the same time, the uncertainty measurement is closely related to the characteristics of deep learning model itself. Therefore, integrating the characteristics of multiple deep learning models as different branches of classifier can effectively improve the robustness of active learning. In order to fully integrate all information of middle hidden layers and consider the advantages of different classification models, we propose an intelligent multi-view active learning method based on double-branch network (MALDB), which can evaluate the uncertainties of samples by jointly considering different branches and different layers of the classifier, so that the most informativeness samples can be selected to improve the performance of deep learning model.

Compared with the existing approaches, our contribution can be summarized as follows: (1) We propose a novel active learning method, which can alleviate the labeling efforts for deep convolutional neural networks; (2) To combine the advantage of different models when selecting unlabeled samples, a double-branch structure with two different Bayesian convolutional neural networks (BCNNs) is introduced into our method. Since each BCNN in the double-branch complete its feature extraction process independently, the characteristics of features obtained by different branches can be effectively integrated to improve the stable of our model; (3) We also adopt a multi-view strategy to leverage multiple level features captured by different hidden layers of network. Through this strategy, a weighted entropy is proposed to estimate the uncertainty of samples. We conduct our experiments on three classical benchmark datasets and two real-world datasets. Experimental results show that our proposed method can improve the performance of the active learning and outperforms other compared approaches.

The paper is organized as follows: Section 2 briefly reviews some related work. Section 3 presents the proposed MALDB. The experimental results on MNIST, Cifar 10, SVHN, Scene–15 and UIUC-Sports datasets are shown and analyzed in Section 4. Finally, Section 5 concludes the paper.

## 2. Related Work

The purpose of active learning is to get a more accurate model with less labeled training data, so that the cost and time of manual annotations can be reduced. In recent years, a lot of work has been put forward to solve this problem. We review the existing work from the following two aspects: active learning based on uncertainty strategy and active learning with multiple views.

### 2.1. Active Learning Based on Uncertainty Criterion

Uncertainty strategy is commonly used in active learning, which measures the uncertainty of candidate unlabeled samples from previous classification predictions. Since it has the great advantage in terms of computational complexity and efficiency, the uncertainty based sample selection strategy works well in combination with some shallow models such as SVM and KNN [22,23]. Tong et al. [22] proposed an active learning method based on a SVM model, which calculates the uncertainty of samples based on the relative distance between the candidate data and decision boundaries. Tuia et al. [24] proposed two variations of active learning models for remote sensing image classification, which can build an optimal set of samples to minimize the classification error. Uncertainty-based sample selection strategies are also widely used in deep learning models. Wang and Shang [14] were the first to apply active learning to deep learning models. They adopted the uncertainty criterion to select samples based on the staked constrained Boltzmann machines and stacked auto-encoders. Gal et al. [25] demonstrated the equivalence between the dropout and approximate Bayesian inference, and proposed an effective method to select the samples with large variance on Bayesian convolutional neural network for label querying. Wang and Zhang [19] tried to query the labels of the most uncertain instances by assigning pseudo labels to instances with higher prediction confidence. Through this way, sufficient labeled data can be obtained for training convolutional neural network. Zhou et al. [26] proposed an active learning method for biomedical image analysis. This method actively optimizes the pre-trained deep neural network by estimating the diversity information among different patches extracted from the same image. Due to the learning progress of shallow models only includes classification output, while the learning progress of deep models contains both feature learning and classification output, the active learning for deep models is different from that for shallow models. However, all of the above uncertainty based active learning methods for deep models only consider the classification output, which neglects a lot of valuable information of different level features learned by intermediate hidden layers. In addition, the selection of samples by only considering the classification output of final layer is very sensitive to the classification result of current classifier [21]. Therefore, in order to better estimate the uncertainty of samples, both the information of intermediate hidden layers and final output layer in the deep learning model should be taken into account.

### 2.2. Active Learning with Multiple Views

The multi-view active learning framework can be traced back to the work of Blum and Mitchell [27], who proposed the concept of “compatibility” between data distribution and target function. Muslea et al. [28] introduced a multi-view active learning method called co-testing, which selects ambiguous data among various views. Yu et al. [29] proposed a method based on Bayesian co-training, which can automatically estimate the different importance of various views. Through theoretically analysis, Wang and Zhou [30] concluded that the samples selected by multi-view active learning are more informative. Zhang and Sun [19] proposed an active learning method for multi-view and multi-learners, in which multiple views are acquired from different learning models. Nevertheless, all above methods are proposed for shallow learning, which cannot be directly applied to deep learning models. In the field of deep learning, Huang et al. [31] proposed an active learning method to estimate the usefulness of samples based on two criteria, which are respectively called distinctiveness and uncertainty. The distinctiveness is obtained by combining the feature information from early to later layers, and the uncertainty of the sample is obtained by combining the maximum entropy. He et al. [21] proposed a multi-view active learning that dynamically combines the uncertainty among hidden layers. The aforementioned two methods combine hidden layer and output layer information to select informative data and achieved good performance. However, the effectiveness of samples selected in them is seriously dependent on the characteristics of a single classifier. Thus, they tend to be sensitive to the ineffectiveness, unstable or bias of the classifier [19]. To mitigate this limitation, multiple classifiers should be combined to select more representative samples [19].

### 2.3. Motivation of Our Work

According to the above review and analysis, the current active learning methods for deep learning framework suffer from the following limitations: First, these methods lose a lot of valuable information since they only take the final output into consideration but ignore the features learned by the middle hidden layers of network. Second, they only adopt a single classifier during the active learning, which may deteriorate their performance when the classifier is ineffective or unstable. These two limitations motivate us to propose a new active learning approach based on multi-view information and double-branch network (i.e., MALDB) to overcome them. To address the first limitation and take full advantage of the information obtained by the network, a multi-view strategy is utilized in our MALDB to fuse the information of different level features from multiple network layers, so that the most uncertain and useful samples can be effectively selected in the process of active learning. Moreover, two different Bayesian convolutional neural networks are employed as the double-branch structure in our approach. The reason for adopting double-branch structure is that different classifiers perform differently on the same sample set in learning and classification process. Therefore, integrating the characteristics of different sub-structures will improve the performance and stability of overall model and overcome the second limitation of the existing methods.

## 3. Multi-View Active Learning Based on Double-Branch Structure

In this section, we will first introduce the structure of our double-branch model, then propose the strategy of sample uncertainty calculation, and at last summarize the main steps of the proposed algorithm.

### 3.1. Double-Branch Network Structure

Deep learning models can effectively learn the representations of samples from generic to specific. Specifically, the first few layers of deep learning models generally capture some basic and common features like shape, color, etc., and the later layers learn more advanced and abstract task-specific features for classification. Therefore, we combine the information of various layers in the network to effectively and intelligently measure the usefulness of samples. Furthermore, in order to overcome the limitation of single branch model, a double-branch network structure is employed in this study to improve the stability of our proposed method.

**Figure 1:**
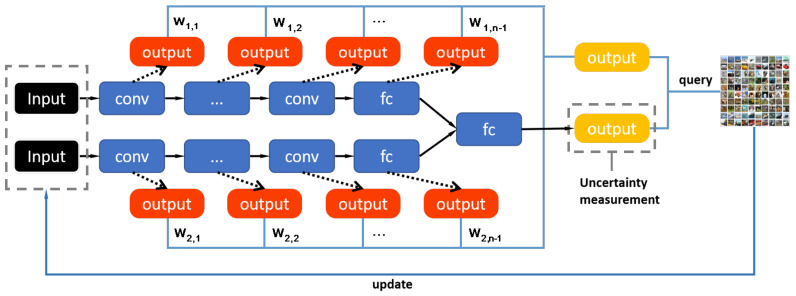
Diagram of our proposed MALDB method. Each ‘outputs’ shown in the figure is used to calculate an uncertainty, and the average of all uncertainty is the final uncertainty score. In the diagram, we only draw one output (shown in yellow box) to calculate the uncertainty for simplicity.

Figure 1 presents the structure of our network. Our main framework is based on two different architectural deep models which are constructed based on Bayesian convolutional neural network. Bayesian convolutional neural network is a CNN with prior probability distributions placed over a set of model parameters $\omega =\{\omega_{1},\ldots ,\omega_{n}\}:\omega \sim p(\omega )$ [25,32]. The reason why we adopt BCNN in our model is that BCNN works well on small batch samples and possesses robustness to over-fitting [32]. Thus, it is more suitable for active learning. Besides, the Bayesian model can improve the performance more rapidly than ordinary convolutional networks, and converge to a higher accuracy [25]. In our study, each Bayesian neural network independently completes its feature extraction process, and their outputs of the last fully connected layer are merged as the final output of overall model. For the feature representations acquired by each convolutional layer of each branch, it is difficult to directly calculate the uncertainty of samples because of its high dimensionality. Therefore, we reshape the high dimensional feature map into a vector and add a softmax layer for each of them. In this way, each convolution layer with an added softmax layer can be considered as an individual entity to calculate its own uncertainty and loss value. The uncertainty indicator of each single entity will participate in the final sample selection, and the loss value will affect the weight of its corresponding uncertainty indicator, but it will not be considered into the back-propagation calculation of the overall model.

### 3.2. Multi-View Sample Selection Strategy

The key of active learning is to develop an effective criterion to measure the value of unlabeled samples. The individual output of each hidden layer is expected to have similar predictions for the same sample in our proposed model. As a result, we utilize the entropy and loss values of all outputs as indicators for sample selection and propose a dynamic multi-level sample selection criterion.

For each hidden layer output, we calculate its uncertainty with respect to a sample using the criterion of max-entropy [14]. Entropy is a commonly used measurement to evaluate the uncertainty of a given sample’s prediction provided by a model. The higher entropy of the sample, the more uncertainty and information the sample has. Hence, the samples with higher entropy should be selected. Assume that the prediction of sample $x_{i}$ obtained by the current output of hidden layer is $p_{i}$, the entropy is defined as:

(1) $$et_{i}=-\sum_{k=1}^{m}p_{i}^{k}\log p_{i}^{k}$$

where *k* denotes the *k*-th candidate of *m* possible labels.

The training progress of our model is continuous and intelligent, that is, the hyperparameters of each layer are constantly optimized through succesive iterations. Thus, it is obviously that the loss calculated from validation dataset is highly related to the feature learned by current hidden layer. Based on the above analysis, we dynamically assign a weight to the entropy of each layer, which can be calculated as follows:

(2) $$w_{i,j}=\frac{e^{-l_{i,j}}}{\sum_{k=1}^{n}e^{-l_{i,k}}}$$

where $w_{i,j}$ is the weight for the entropy of the *j*-th hidden layer output in the *i*-th branch, $l_{i,j}$ is the loss of *j*-th softmax layer in the *i*-th branch evaluated by the validation dataset.

In Equation (2), each weight represents the current hidden layer’s contribution to overall uncertainty. Based on multiple experiments, we found that the smaller the loss, the greater the contribution of this hidden layer to the overall selection process. Therefore, we defined the weighted entropy as follows:

(3) $$En_{i}=\sum_{j=1}^{n-1}w_{i,j}\cdot et_{i,j}$$

where $En_{i}$ is the combined entropy of *i*-th branch. $et_{i.j}$ is the entropy of *j*-th softmax layer in the *i*-th branch, which is calculated by Equation (1).

Finally, the uncertainty of our proposed strategy for selecting samples is defined as follows:

(4) $$score=\frac{w_{1,n-1}}{w_{1,n-1}+w_{2,n-1}}En_{1}+\frac{w_{2,n-1}}{w_{1,n-1}+w_{2,n-1}}En_{2}+et_{n}$$

where the first two terms are the normalized weighted entropy of two branches and $et_{n}$ is the entropy obtained by the final output of entire model.

In Equation (4), both the information of hidden layers and final output of the network is combined as an indicator to measure the uncertainty of the sample. The sample with high score will be taken out to query their labels and incorporated into the training set for the next round of training.

### 3.3. Overall Algorithm

We provide the implementation scheme of our method in Algorithm 1.
**Algorithm 1.** Multi-view active learning based on double-branch networkInput:   ***X^l^***, ***X^u^***, ***M_0_***, ***n****, **ƒ***, ***R***, ***T***, ***O_i,j_*** {***X^l^*** is initial labeled dataset; ***X^u^*** is unlabeled data; ***M_0_***
*is* initial model;  ***n***
*is* the number of softmax layers; ***ƒ*** is calculate the entropy of output using Equation (1); ***R*** is  the number of unlabeled samples to be queried in each iteration; ***T*** is the total iteration  number of the query; ***O_i,j_*** is output of the hidden layer}**Initialization:****L_0_ = X^l^, U_0_ = X^u^**Divide *L_0_* into two parts: randomly initial training dataset *L_train_* and validation dataset *L_valid_*  1: **for**
*i* = 0 … *T-1*
**do**  2:  add softmax layer to each hidden layer of each branch in *M_0_*
  3:  *M_i+1_* = train(*M_i_, L_train_*)  4:   **for**
*j* = 1 … *n*-1 **do**  5:    compute loss *l*_1, *j*_, *l*_2,*j*_ of each hidden layer in each branch by using *L_valid_*
  6:    compute $w_{1,j}=\frac{e^{-l_{1,j}}}{\sum_{k=1}^{n-1}e^{-l_{1,k}}}$ and $w_{2,j}=\frac{e^{-l_{2,j}}}{\sum_{k=1}^{n-1}e^{-l_{2,k}}}$ using Equation (2)  7:   **end for**  8:   **for**
*x^add^*… *U_i_*
**do**  9:    compute *score* using Equations (3)–(4):$score^{1,add}=\sum_{i=1}^{n-1}w_{1,i}\cdot f(O_{1,i}(x^{add}))$, $score^{2,add}=\sum_{i=1}^{n-1}w_{2,i}\cdot f(O_{2,i}(x^{add}))$
  $score^{final}=score^{1,add}\cdot \frac{w_{1,n-1}}{w_{1,n-1}+w_{2,n-1}}+score^{2,add}\cdot \frac{w_{2,n-1}}{w_{1,n-1}+w_{2,n-1}}+f(O^{final}(x^{add}))$
  10:   **end for**  11:   Label the *R* instances with largest *score* in *U_i_* to form *Q_i_*  12:   update *L_i+1_* = *L_i_*∪*Q_i_* and *U_i+1_ = U_i_* – *Q_i_*
  13: **end for****Output:*****M_T-1_***: the final trained model

## 4. Experiments

In this section, we evaluate our proposed approach on different datasets and compare its performance with the baselines and other algorithms. All experiments are implemented in Python with Keras.

### 4.1. Datasets and Experimental Setup

#### 4.1.1. Datasets

**Figure 2:**
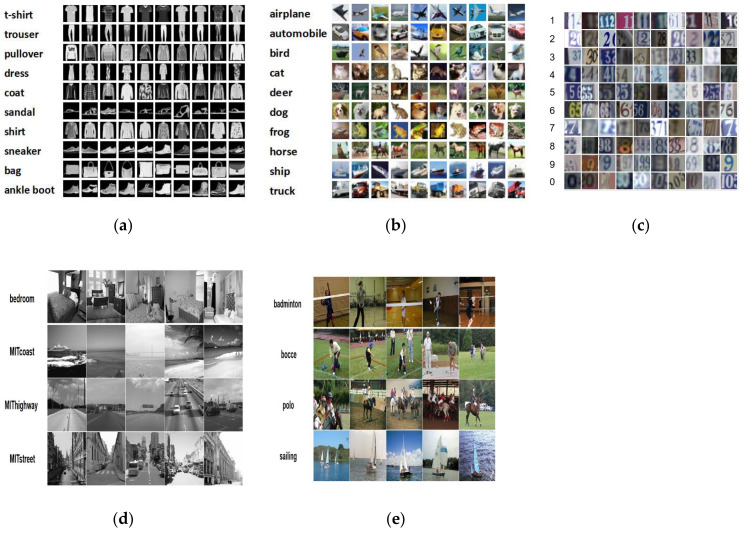
Example images of different datasets. (**a**) Fashion-MNIST [33], (**b**) CIFAR-10 [34], (**c**) SVHN [35], (**d**) Scene-15 [36], (**e**) UIUC-Sports [37].

Our proposed approach is evaluated on three classical benchmark datasets, Fashion-MNIST [33], CIFAR-10 [34] and SVHN [35], which are widely used for active learning tasks. Furthermore, two real-world datasets (scene-15 [36] and UIUC-Sports [37]) for scene classification tasks were also utilized to test the performance of our MALDB. The Fashion-MNIST dataset consists of 70,000 gray images that are labeled as 10 everyday wear categories like t-shirts, trousers and so on. The resolution of each image is 28 × 28. The Fashion-MNIST dataset has been officially split into 60,000 training images and 10,000 testing images, respectively. The Cifar-10 includes 60,000 color images with 10 complex categories, which has been officially divided into 50,000 training images and 10,000 testing images. The resolution of each image in Cifar-10 dataset is 32 × 32. The SVHN dataset is obtained from house numbers in Google Street View images. There are 73,257 RGB images for training and 26,032 images for testing. All digits in SVHN have been resized to a fixed resolution of 32 × 32. The Scene-15 dataset [36] consists of 15 scene categories with a total of 4485 images, which are approximately 300 × 250 in average resolution. In this experiment, we resize the resolution of images in this dataset as 200 × 200. The UIUC-Sports dataset [37] contains 1585 images of eight sports scene classes, and the minimum resolution of the images is about 800 × 600. We resize the resolution of images in this dataset as 400 × 400 in our experiment. Figure 2 shows example images of these five datasets.

#### 4.1.2. Experimental Setup

##### Models

For the Fashion-MNIST dataset, we made some minor changes based on LeNet architecture [38], and merged it with the Bayesian CNN mentioned in [25]. The details of each branch structure in our double-branch network are: (a) Branch-1: convolution-relu-maxpooling-dropout- convolution- relu-maxpooling-dropout-convolution-dense-dropout-dense-softmax, (b) Branch-2: convolution- relu-convolution-relu-maxpooling-dropout-dense-relu-dropout-dense-softmax, with 32 convolution kernels, 4 × 4 kernel size, 2 × 2 pooling, dense layer with 128 units, and dropout probabilities are set to 0.25 and 0.5. For the Cifar-10, SVHN, Scene-15 and UIUC-Sports datasets, we replaced the LeNet architecture with the model in [21].

##### Hyper Parameter

In our experiments, the initial labeled training samples for training our model are completely randomly selected. To reduce the interference of randomness, when we compare our proposed method with other approaches, we ensure that the same initial labeled data are input into them. Specifically, we randomly select 10% of training data as the validation set, and then randomly choose 1000 samples from the rest training data as the initial labeled data to train the models. The remaining samples are regarded as unlabeled data pool. The number of iterations of sample selection process is set as 150. At each iteration, the weights of the best validation accuracy in all epochs will be saved and *q* samples will be queried from the unlabeled data pool to join the training set. Then the best test accuracy of various models is reported. For Fashion-MNIST dataset, we set *q* as 100. For Cifar10 and SVHN, *q* is set as 200. For Scene-15 and UIUC-Sports, the images are randomly split into labeled training set, unlabeled set and testing set according to proportions of 10%, 60% and 30%, respectively. The parameter *q* is set as 200 and 100 samples for UIUC-Sports and Scene-15 datasets. The maximum number of iterations is set to 10 for the Scene-15, while it is set to 8 for the UIUC-Sports dataset because the number of samples in this dataset is small. The SGD optimizer with learning rate 0.001 and momentum 0.9 is employed to optimize our model. We set the batch size as 32 and set max epoch as 50 with early stopping. In this study, 100 sets of parameters (i.e., *ω* in BCNN) are sampled from the model parameter distribution for each forward pass. No data augmentation is used during training.

##### Environment

Our experiments are performed on a machine with a single graphics card (NVIDIA GTX 1080Ti), a six-core Intel i7 processor and 16 Gb memory.

##### Baselines

To prove that our proposed model and sample selection measurement are effective we compare our method (MALDB for short) with the following baselines: selecting samples randomly (our model-RAND for short) and full data training (ALL for short). The above two baselines utilize the double-branch BCNN as their backbone networks, which is the same as our proposed MALDB. Besides, we also compare the performance of our approach with other existing methods including: max-entropy selection strategy based on Bayesian CNN (BCNN-EN for short) [25], active learning with multiple views (AL-MV for short) [21] and standard CNN with random sample selection (CNN for short) [3].

### 4.2. Experimental Results and Analysis

In this section, we present the classification results on five datasets to demonstrate the effectiveness of our active learning algorithm. In order to reduce the deviation caused by randomness, we repeat the experiments five times to obtain the average test accuracy, standard deviation, precision, recall and F1-score of different methods.

Table 1 lists the average test accuracy and standard deviation of each method on Fashion-MNIST dataset when selecting 100, 5000, 10,000 and 15,000 samples. Table 2 and Table 3 show the results on Cifar-10 and SVHN datasets when selecting 200, 10,000, 20,000 and 30,000 samples, respectively. Table 4 shows the results on Scene-15 dataset when selecting 400, 800, 1200, 1600 and 2000 samples. Table 5 shows the results on UIUC-Sports dataset when selecting 200, 400, 600, 800 samples.

From these tables, we can find that the performance of MALDB is generally superior to that of the other methods. Furthermore, it can be seen that though only 22.86%, 51.67%, 42.32%, 54.58% and 60.60% of training data in Fahsion-MNIST, Cifar-10, SVHN, Scene-15 and UIUC-Sports datasets is selected by the proposed method for training, the classification accuracy obtained by our MALDB is very close to the results obtained by the entire training sets (ALL), which indicates that our method can effectively find sample subsets which provide nearly the same information as the entire datasets.

**Figure 3:**
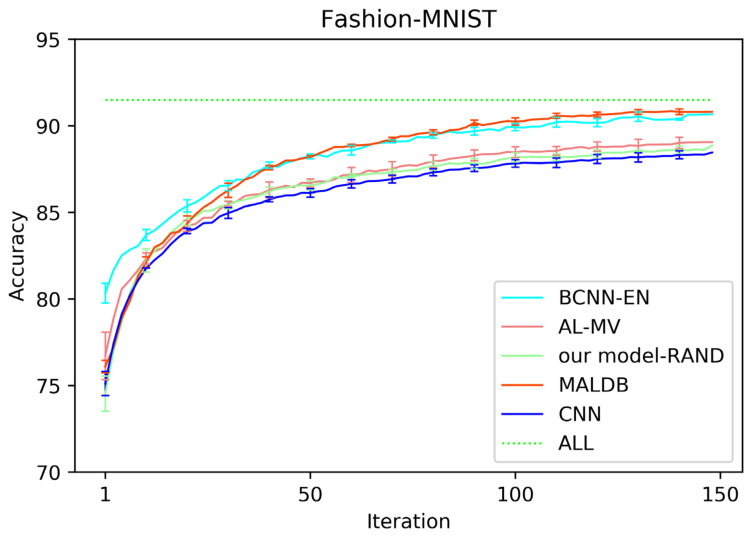
Test accuracy curve of different methods on Fashion-MNIST dataset.

**Figure 4:**
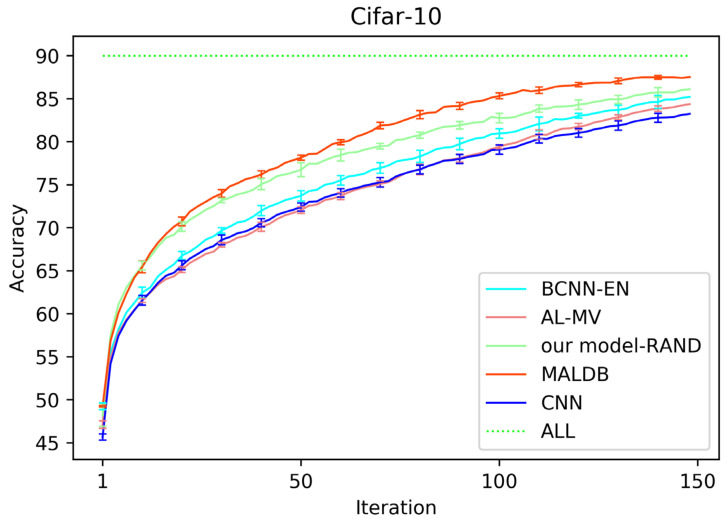
Test accuracy and standard deviation curve of different methods on Cifar-10 dataset.

**Figure 5:**
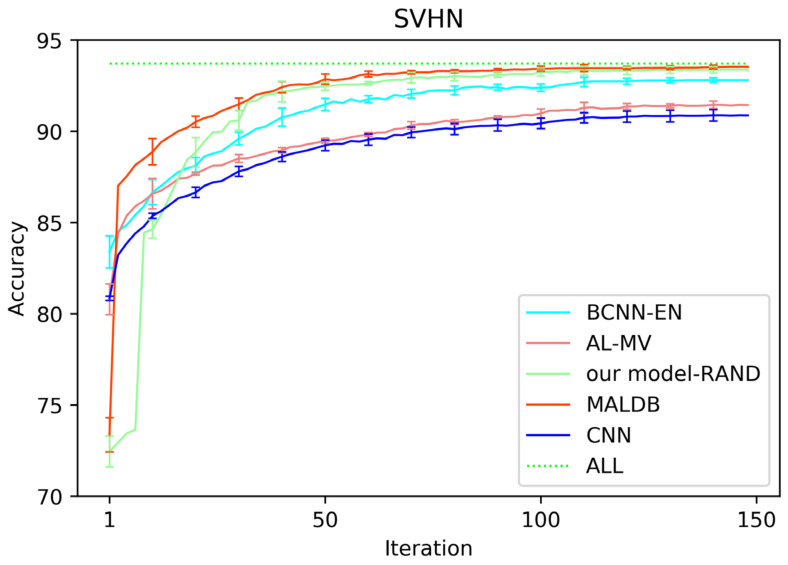
Test accuracy and standard deviation curve of different methods on SVHN dataset.

**Figure 6:**
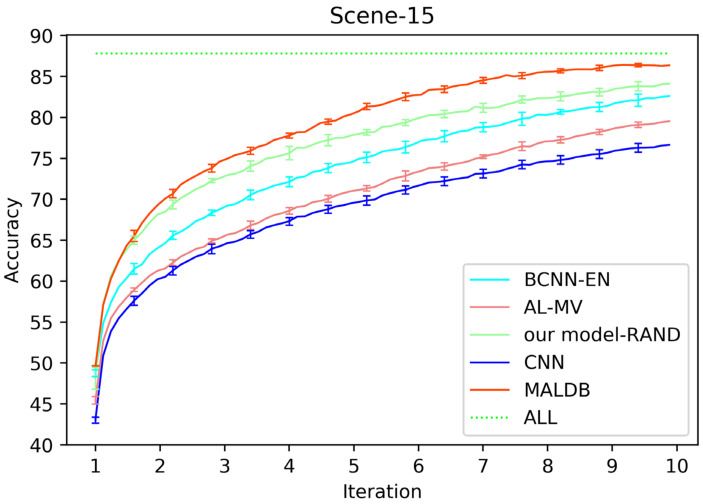
Test accuracy and standard deviation curve of different methods on Scene-15 dataset.

**Figure 7:**
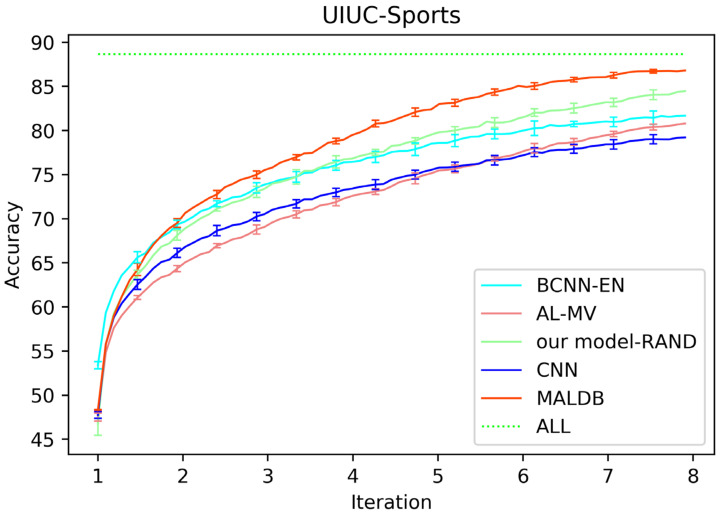
Test accuracy and standard deviation curve of different methods on UIUC-Sports dataset.

Figure 3, Figure 4, Figure 5, Figure 6 and Figure 7 show the average test accuracy curves of different methods under different number of query iterations on five datasets. Combining the information of these results, we can get the following observations. First, due to the network structures of BCNN-EN and AL-MV are one branch and the number of parameters needed to be optimized in them is less than our method, they have a better ability to capture feature information than our double-branch model when the amount of training data is small. Thus, their performance is better than the proposed MALDB in the first few iterations. This phenomenon is particularly evident for SVHN and UIUC-Sports since these datasets are more complex. Nevertheless, with the increase in the number of iterations, our MALDB outperforms BCNN-EN and AL-MV rapidly, which indicates our model can better remove interference information in a short time and capture useful information. Second, the classification accuracy obtained by our MALDB is superior to random sample selection strategy (our model-RAND) on all datasets. This result demonstrates that the active learning can effectively select the most informative samples to improve the performance of our model. Third, the advantage of our MALDB over standard CNN with random sample selection (referred as CNN) can also show the effectiveness of active learning mechanism and double-branch structure in our approach. At last, we can find the standard deviations obtained by our proposed MALDB are less than other approaches on all datasets, which justifies that the double-branch network structure in our model can reduce the performance fluctuation and improve the stability of active learning.

Here, it should be noted that since the within-class scatter of samples in Cifar-10, scene-15 and UIUC-Sports datasets is high, the accuracy obtained by all methods is relatively low (less than 90%). However, our MALDB still outperforms other approaches in these three datasets, which indicates the proposed active learning and sample selection mechanisms are effective.

Then, the precision and recall are adopted as two measurements to evaluate the performance of our MALDB. For the *i*-th class, its precision and recall can be obtained by:

(5) $$precision=\frac{TP_{i}}{TP_{i}+FP_{i}}$$

(6) $$recall=\frac{TP_{i}}{TP_{i}+FN_{i}}$$

where *TP_i_* is the number of samples that belong to the *i*-th class and are correctly classified, *FP_i_* is the number of cases that don’t belong to the *i*-th class but are incorrectly classified as belonging to this class, *FN_i_* is the number of cases that belong to the *i*-th class but are incorrectly classified as belonging to other classes.

**Figure 8:**
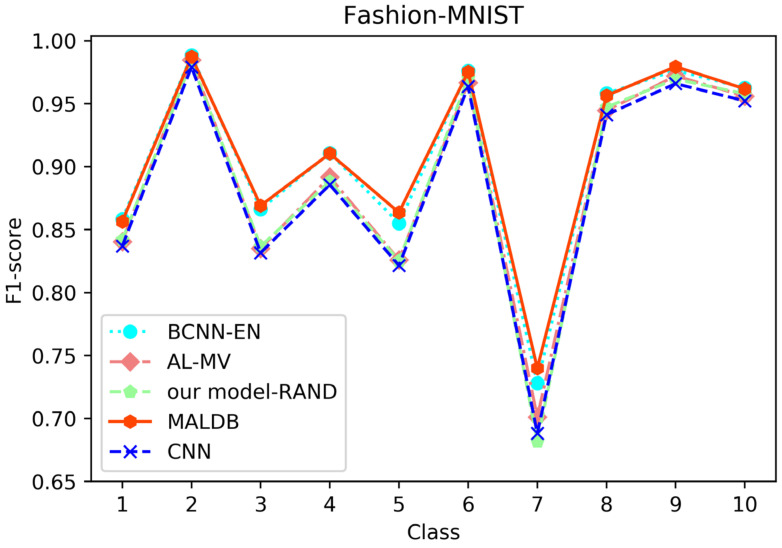
F1-score of the 150th iteration obtained by different methods on Fashion-MNIST dataset.

**Figure 9:**
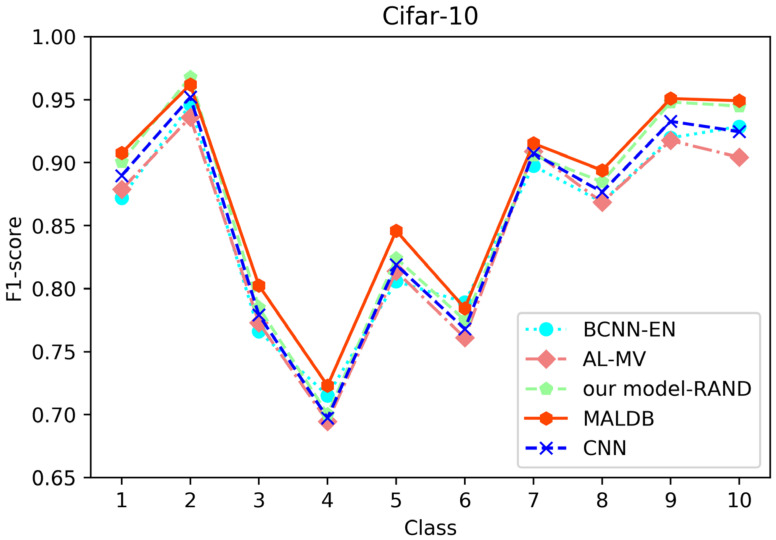
F1-score of the 150th iteration obtained by different methods on Cifar-10 dataset.

**Figure 10:**
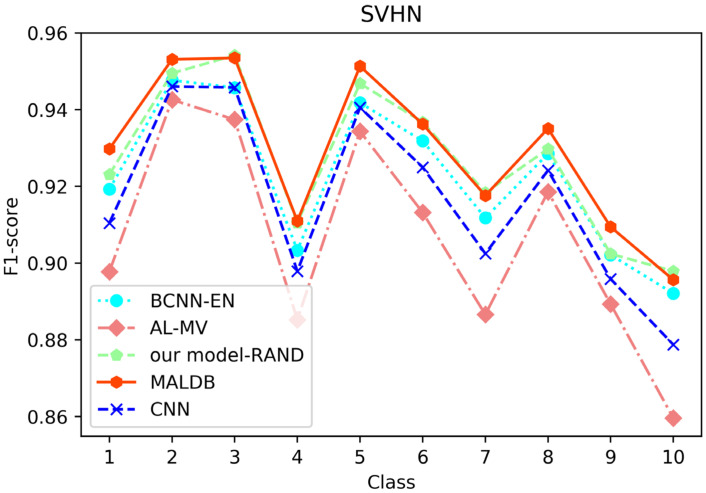
F1-score of the 150th iteration obtained by different methods on SVHN dataset.

**Figure 11:**
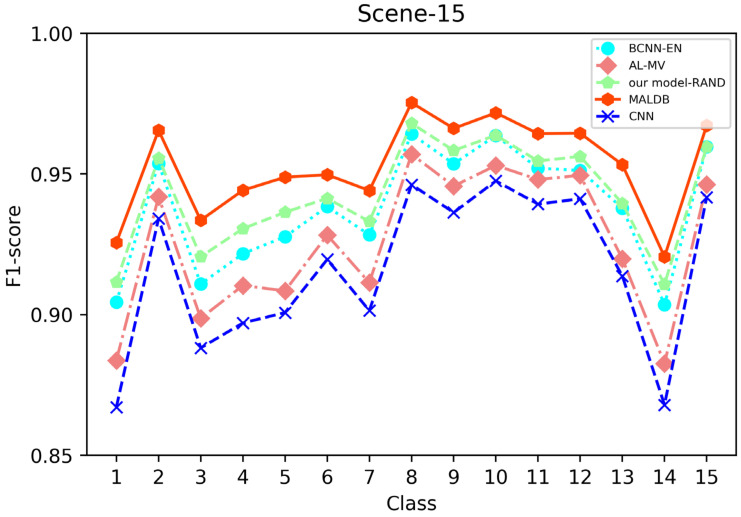
F1-score of the 10th iteration obtained by different methods on Scene-15 dataset.

**Figure 12:**
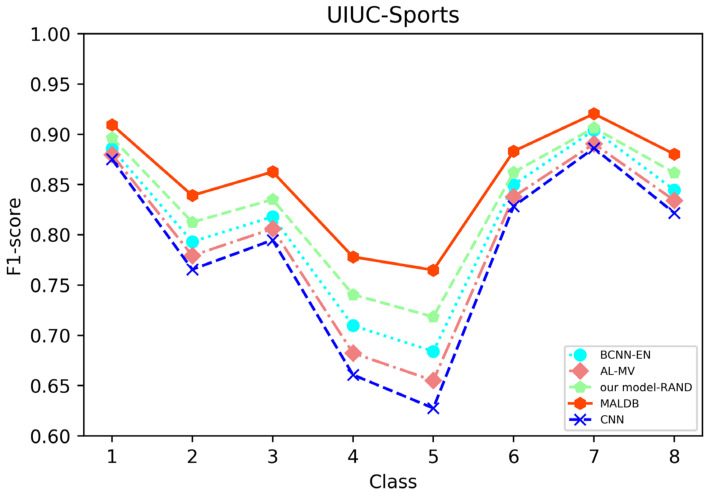
F1-score of the 8th iteration obtained by different methods on UIUC-Sports dataset.

From the average precision and recall of all classes after the last iteration obtained by each method in Table 6, Table 7, Table 8, Table 9 and Table 10, it can be seen that our MALDB outperforms other approaches. In addition, the F1-score, which is a harmonic mean of precision and recall, is also employed in our experiment to further compare the performance of different approaches. From the F1-score of each class obtained by various methods in Figure 8, Figure 9, Figure 10, Figure 11 and Figure 12, it can be seen that our MALDB is superior to other approaches in most cases. The average F1-score of all classes on five datasets in Table 11 also demonstrates the advantage of the proposed method.

Next, the computational complexity of the proposed MALDB is analyzed. In deep learning- based models, the computational complexity is closely related to the number of parameters needed to be optimized in it. Thus, we first tabulate the number of parameters in different methods in Table 12. Then, the average time of each epoch in training different methods is shown in Table 13. From this table, we can find that the computational complexity in the training process of the proposed MALDB is higher than other methods. This is due to the following two reasons. First, the double-branch structure in our MALDB contains more parameters than other approaches. Thus, it needs more time to optimize them. Second, the proposed MALDB estimate the uncertainty of each sample by combining multi-view information to calculate the weighted entropy, which also increases the training time. Nevertheless, from Table 13, it also can be seen that the average test time for classifying each sample of our MALDB is not much longer than other methods, which means the proposed method is executable.

**Figure 13:**
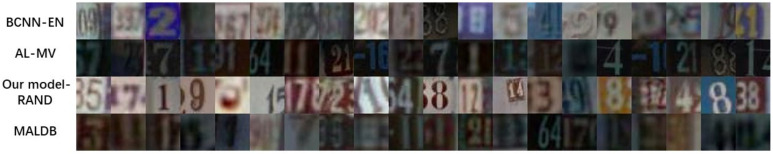
The images with the largest uncertainty selected by different methods on SVHN dataset.

To visually compare different approaches, 20 images of SVHN dataset with the largest uncertainty selected by different methods after the first iteration are shown in Figure 13. We can see the samples selected by our MALDB are more ambiguous than those selected by other methods. That is, they are either difficult to distinguish from background or contain more than one numbers in the picture. Thus, incorporating these informative samples into the training set will help to improve the performance of the model. Moreover, the informative samples selected by our approach are consistent with human’s intuition to some extent. In other words, some images selected by MALDB are also unclear for us.

### 4.3. Ablation Experiment

**Figure 14:**
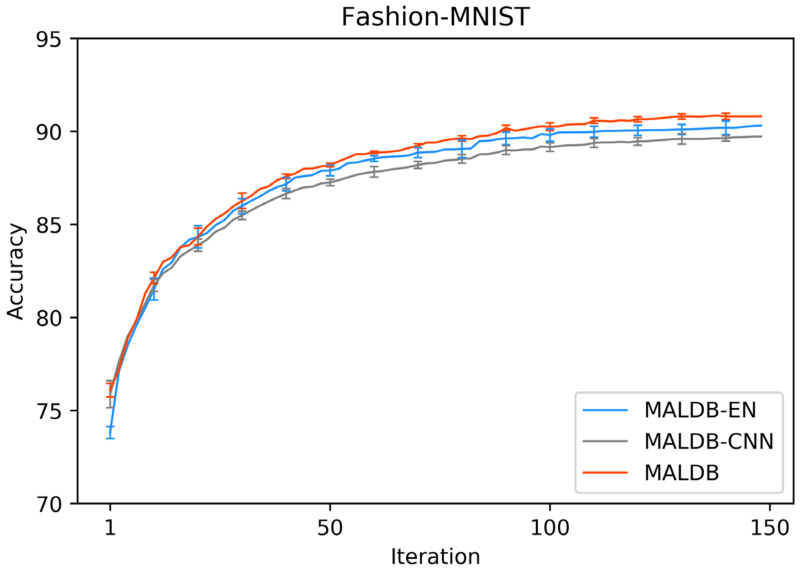
Test accuracy curve of ablation experiment on Fashion-MNIST dataset.

**Figure 15:**
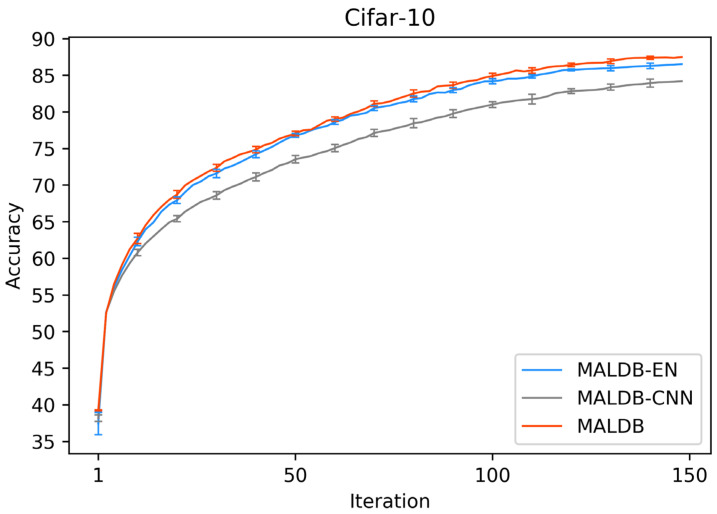
Test accuracy curve of ablation experiment on Cifar-10 dataset.

**Figure 16:**
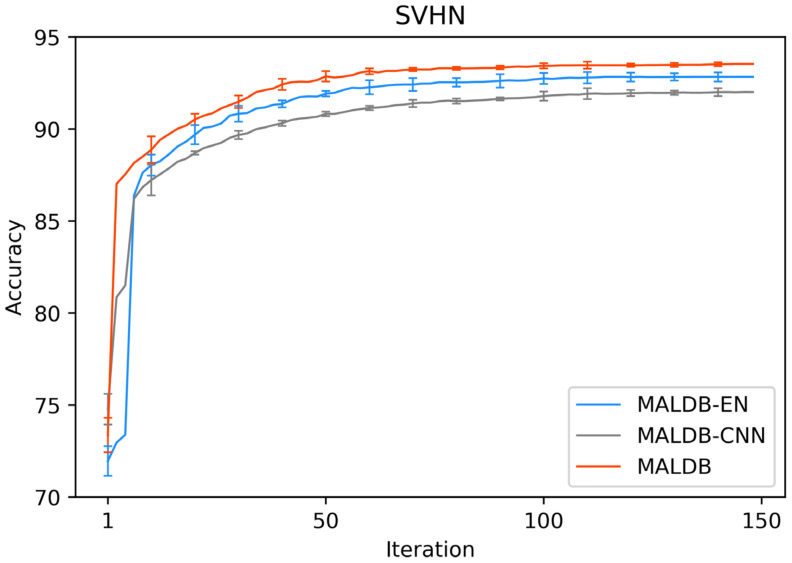
Test accuracy curve of ablation experiment on SVHN dataset.

**Figure 17:**
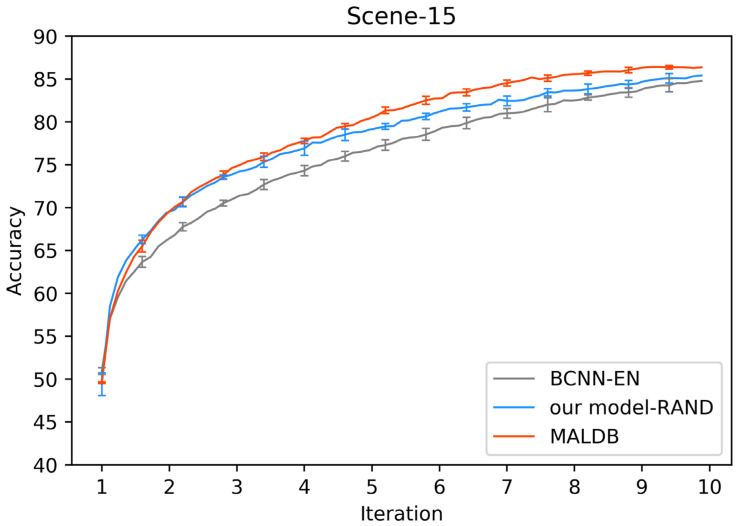
Test accuracy curve of ablation experiment on Scene-15 dataset.

**Figure 18:**
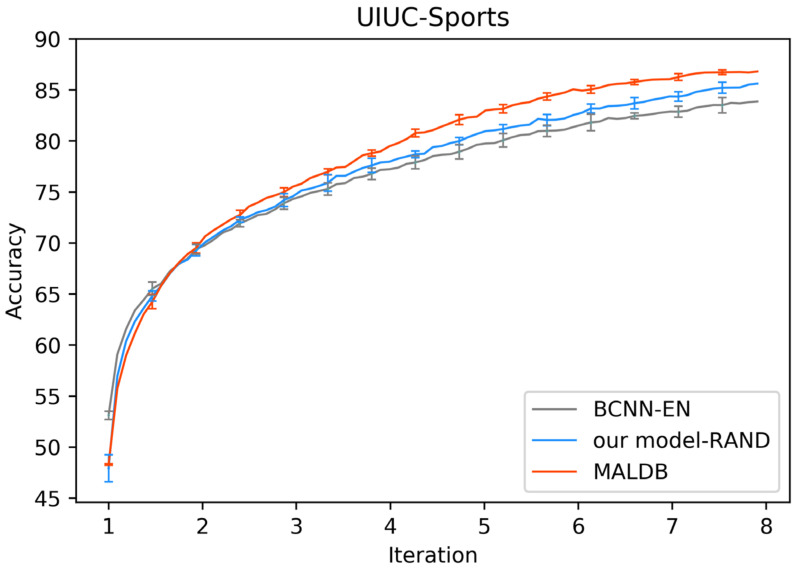
Test accuracy curve of ablation experiment on UIUC-Sports dataset.

In order to justify the multi-view information and BCNN utilized in our method, two ablation experiments are conducted in this subsection. In the first ablation experiment, we compare the performance of our MALDB with the same model without multi-view information (referred to as ‘MALDB-EN’). MALDB-EN neglects the information of middle hidden layers in the network and selects the samples only based on the information of final output. In the second ablation experiment, we replace the BCNN in our model with the standard CNN (referred to as ‘MALDB-CNN’). From the experimental results in Figure 14, Figure 15, Figure 16, Figure 17 and Figure 18 and Table 14, Table 15, Table 16, Table 17 and Table 18, we can find that our MALDB outperforms MALDB-EN and MALDB-CNN, which means both the multi-view and BCNN are essential for our method to improve the performance.

**Figure 19:**
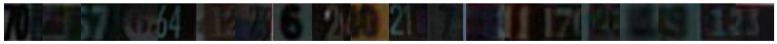
Images with contrary uncertainty from the SVHN dataset.

Finally, for the sake of demonstrating the impact of entropy obtained by different intermediate layer outputs on the selected samples, some images from SVHN dataset, which have low uncertainty on the final outputs but high uncertainty on the intermediate layers, are shown in Figure 19. From this figure, it can be found that most of these images have two or one and a half numbers. Therefore, though the intermediate layers of the network can capture some useful features of the numbers in these images, the final outputs of the network will still be confused.

## 5. Conclusions

In this paper we propose an intelligent multi-view active learning method based on a double-branch network for image classification tasks. The proposed method employs two BCNNs with different architecture and adopts a dynamic multi-view sample selection strategy to select informative samples. Extensive experiments were performed on three commonly used datasets, Fashion-MNIST, Cifar-10, SVHN, Scene-15 and UIUC-Sports. The experimental result illustrates that our method achieves better performance than other approaches.

At last, it should be pointed out that although we only utilized the image datasets to evaluate the performance of our MALDB in this study, the application of our proposed approach is not restricted to image classification tasks. For example, through replacing the 2D convolution kernel in BCNN with a 1D or 3D convolution kernel, our MALDB can be applied to natural language processing or video analysis problem. Thus, one of our future tasks will be to apply the proposed model to other research fields so that it can be more widely used. Besides, another direction of our future study is to introduce some more state-of-the-art techniques (such as attention mechanisms [39], graph neural networks [40] and Res-Net [41]) into MALDB to test their impact on our model and try to further improve its effectiveness and flexibility.

## Figures and Tables

**Table 1:** Test accuracy (%) ± standard deviation (%) obtained by each method under different number of query iterations on Fashion-MNIST.

	Methods	1	50	100	150
Iteration					
**BCNN-EN**		80.334 ± 0.570	88.130 ± 0.265	89.930 ± 0.203	90.662 ± 0.217
**AL-MV**		76.712 ± 1.369	86.768 ± 0.301	88.502 ± 0.327	89.022 ± 0.262
**Our model-RAND**		74.532 ± 1.021	86.528 ± 0.253	88.180 ± 0.260	88.850 ± 0.427
**CNN**		76.096 ± 0.362	88.194 ± 0.198	90.212 ± 0.193	90.812 ± 0.144
**MALDB**		75.122 ± 0.353	86.137 ± 0.085	87.804 ± 0.256	88.461 ± 0.243
**ALL**		91.500 ± 0.320	91.500 ± 0.320	91.500 ± 0.320	91.500 ± 0.320

**Table 2:** Test accuracy (%) ± standard deviation (%) obtained by each method under different number of query iterations on Cifar-10.

	Methods	1	50	100	150
Iteration					
**BCNN-EN**		43.418 ± 0.401	72.787 ± 0.652	80.625 ± 0.363	85.204 ± 0.228
**AL-MV**		41.381 ± 0.453	71.069 ± 0.484	78.911 ± 0.353	84.356 ± 0.352
**Our model-RAND**		38.236 ± 1.319	75.310 ± 0.732	82.464 ± 0.477	86.098 ± 0.314
**CNN**		45.683 ± 0.371	72.087 ± 0.363	79.022 ± 0.237	83.238 ± 0.313
**MALDB**		39.242 ± 0.085	76.712 ± 0.566	84.704 ± 0.427	87.496 ± 0.188
**ALL**		90.020 ± 0.170	90.020 ± 0.170	90.020 ± 0.170	90.020 ± 0.170

**Table 3:** Test accuracy (%) ± standard deviation (%) obtained by each method under different number of query iterations on SVHN.

	Methods	1	50	100	150
Iteration					
**BCNN-EN**		83.395 ± 0.877	91.313 ± 0.357	92.368 ± 0.181	92.804 ± 0.129
**AL-MV**		80.794 ± 0.837	89.377 ± 0.071	90.884 ± 0.186	91.443 ± 0.163
**Our model-RAND**		73.492 ± 0.817	92.133 ± 0.281	93.140 ± 0.165	93.273 ± 0.209
**CNN**		80.844 ± 0.324	89.094 ± 0.253	90.376 ± 0.281	90.873 ± 0.266
**MALDB**		73.363 ± 0.934	92.657 ± 0.346	93.413 ± 0.133	93.487 ± 0.103
**ALL**		93.723 ± 0.523	93.723 ± 0.523	93.723 ± 0.523	93.723 ± 0.523

**Table 4:** Test accuracy (%) ± standard deviation (%) obtained by each method under different number of query iterations on Scene–15.

	Methods	2	4	6	8	10
Iteration						
**BCNN-EN**		68.766 ± 0.321	74.524 ± 0.562	78.744 ± 0.365	81.393 ± 0.268	82.612 ± 0.265
**AL-MV**		65.234 ± 0.413	70.991 ± 0.674	74.961 ± 0.535	78.429 ± 0.478	79.534 ± 0.301
**Our model-RAND**		72.563 ± 0.619	77.808 ± 0.522	81.312 ± 0.625	83.190 ± 0.236	84.121 ± 0.253
**CNN**		64.248 ± 0.417	69.522 ± 0.733	73.103 ± 0.423	75.673 ± 0.573	76.647 ± 0.198
**MALDB**		74.602 ± 0.266	80.392 ± 0.386	84.349 ± 0.465	86.217 ± 0.320	86.360 ± 0.085
**ALL**		87.82 ± 0.233	87.82 ± 0.233	87.82 ± 0.233	87.82 ± 0.233	87.82 ± 0.233

**Table 5:** Test accuracy (%) ± standard deviation (%) obtained by each method under different number of query iterations on UIUC-Sports.

	Methods	2	4	6	8
Iteration					
**BCNN-EN**		69.339 ± 0.674	76.442 ± 0.522	79.876 ± 0.417	81.682 ± 0.625
**AL-MV**		64.352 ± 0.365	72.575 ± 0.321	77.454 ± 0.535	80.790 ± 0.478
**Our model-RAND**		68.125 ± 0.733	76.802 ± 0.562	81.312 ± 0.268	84.468 ± 0.573
**CNN**		66.125 ± 0.413	73.40 ± 0.619	77.065 ± 0.386	79.225 ± 0.236
**MALDB**		69.523 ± 0.465	79.475 ± 0.423	85.067 ± 0.266	86.816 ± 0.320
**ALL**		88.68 ± 0.414	88.68 ± 0.414	88.68 ± 0.414	88.68 ± 0.414

**Table 6:** Average recall and precision of all classes obtained by each method after the 150th iteration on Fashion-Mnist dataset.

	Methods	BCNN-EN	AL-MV	Our Model-RAND	CNN	MALDB
Evaluate						
**Recall**		0.9079	0.8918	0.8903	0.8909	0.9098
**Precision**		0.9083	0.8924	0.8902	0.8913	0.9102

**Table 7:** Average recall and precision of all classes obtained by each method after the 150th iteration on Cifar-10 dataset.

	Methods	BCNN-EN	AL-MV	Our Model-RAND	CNN	MALDB
Evaluate						
**Recall**		0.8509	0.8450	0.8632	0.8541	0.8733
**Precision**		0.8523	0.8472	0.8655	0.8564	0.8747

**Table 8:** Average recall and precision of all classes obtained by each method after the 150th iteration on SVHN dataset.

	Methods	BCNN-EN	AL-MV	Our Model-RAND	CNN	MALDB
Evaluate						
**Recall**		0.9230	0.9070	0.9272	0.9171	0.9293
**Precision**		0.9221	0.9061	0.9269	0.9165	0.9295

**Table 9:** Average recall and precision of all classes obtained by each method after the 10th iteration on Scene-15 dataset.

	Methods	BCNN-EN	AL-MV	Our Model-RAND	CNN	MALDB
Evaluate						
**Recall**		0.9370	0.9249	0.9420	0.9153	0.9524
**Precision**		0.9369	0.9272	0.9439	0.9180	0.9540

**Table 10:** Average recall and precision of all classes obtained by each method after the 8th iteration on UIUC-Sports dataset.

	Methods	BCNN-EN	AL-MV	Our Model-RAND	CNN	MALDB
Evaluate						
**Recall**		0.8143	0.7961	0.8296	0.7828	0.8554
**Precision**		0.8219	0.8005	0.8319	0.7881	0.8565

**Table 11:** The average F1-score obtained by each method on five datasets.

	Dataset	Fahsion-MNIST	CIFAR-10	SVHN	Scene-15	UIUC-Sports
Methods						
**BCNN-EN**		0.9080	0.8515	0.9225	0.9369	0.8180
**AL-MV**		0.8920	0.8460	0.9065	0.9260	0.7982
**Our model-RAND**		0.8902	0.8643	0.9270	0.9429	0.8307
**CNN**		0.8910	0.8552	0.9167	0.9166	0.7854
**MALDB**		0.9099	0.8739	0.9293	0.9531	0.8559

**Table 12:** The number of parameters in each model on different datasets. (m indicates million).

	Dataset	Fahsion-MNIST	CIFAR-10	SVHN	Scene-15	UIUC-Sports
Methods						
**BCNN-EN**		0.149 m	0.191 m	0.191 m	16.706 m	74.050 m
**AL-MV**		0.302 m	1.770 m	1.770 m	121.225 m	514.262 m
**Our model-RAND**		0.548 m	2.550 m	2.550 m	173.3 m	735.869 m
**CNN**		0.302 m	1.770 m	1.770 m	121.225 m	514.262 m
**MALDB**		0.548 m	2.550 m	2.550 m	173.3 m	735.869 m

**Table 13:** Average time of each epoch in training and test time for classifying each sample of different methods.

		Dataset				
	Avg. Epoch Time/Test Time	Fashion-MNIST	CIFAR-10	SVHN	Scene-15	UIUC-Sports
Methods						
**BCNN-EN**		36.1s/0.201s	42.0s/0.219s	42.0s/0.219s	556.0s/0.351s	276.3s/0.498s
**AL-MV**		58.9s/0.307s	74.3s/0.387s	74.3s/0.387s	824.1s/0.511s	488.8s/0.865s
**Our model-RAND**		128.3s/0.611s	132.5s/0.631s	132.5s/0.631s	1655.8s/0.880s	990.6s/1.229s
**CNN**		58.9s/0.307s	78.5s/0.374s	78.5s/0.374s	824.1s/0.511s	488.8s/0.865s
**MALDB**		128.3s/0.611s	145.9s/0.695s	145.9s/0.695s	1655.8s/0.880s	990.6s/1.229s

**Table 14:** Test accuracy (%) ± standard deviation (%) obtained by various methods under different number of query iterations on Fashion-MNIST dataset.

	Methods	1	50	100	150
Iteration					
**MALDB-EN**		74.014 ± 0.320	87.840 ± 0.347	89.974 ± 0.323	90.512 ± 0.211
**MALDB-CNN**		75.882 ± 0.728	87.253 ± 0.264	89.155 ± 0.204	89.888 ± 0.217
**MALDB**		76.096 ± 0.362	88.194 ± 0.198	90.212 ± 0.193	90.812 ± 0.144

**Table 15:** Test accuracy (%) ± standard deviation (%) obtained by various methods under different number of query iterations on Cifar-10 dataset.

	Methods	1	50	100	150
Iteration					
**MALDB** **-EN**		37.452 ± 0.437	76.388 ± 0.398	84.166 ± 0.560	86.542 ± 0.474
**MALDB-CNN**		38.183 ± 1.541	72.997 ± 0.479	80.782 ± 0.319	84.194 ± 0.307
**MALDB**		39.242 ± 0.085	76.712 ± 0.566	84.704 ± 0.427	87.496 ± 0.188

**Table 16:** Test accuracy (%) ± standard deviation (%) obtained by various methods under different number of query iterations on SVHN dataset.

	Methods	1	50	100	150
Iteration					
**MALDB** **-EN**		71.848 ± 0.806	91.875 ± 0.174	92.655 ± 0.248	92.773 ±0.182
**M** **AL** **DB-CNN**		74.735 ± 0.809	90.808 ±0.346	91.756 ± 0.287	91.984 ±0.230
**MALDB**		73.363 ± 0.934	92.657 ±0.533	93.413 ± 0.133	93.487 ± 0.103

**Table 17:** Test accuracy (%) ± standard deviation (%) obtained by various methods under different number of query iterations on Scene-15 dataset.

	Methods	2	4	6	8	10
Iteration						
**MALDB** **-EN**		73.843 ± 0.605	79.088 ± 0.103	82.592 ± 0.230	84.470 ± 0.533	85.401 ± 0.133
**M** **AL** **DB-CNN**		70.939 ± 0.558	76.698 ± 0.230	80.917 ± 0.103	83.566 ± 0.346	84.785 ± 0.248
**MALDB**		74.602 ± 0.714	80.392 ± 0.182	84.349 ± 0.182	86.217 ± 0.174	86.360 ± 0.287

**Table 18:** Test accuracy (%) ± standard deviation (%) obtained by various methods under different number of query iterations on UIUC-Sports dataset.

	Methods	2	4	6	8
Iteration					
**MALDB** **-EN**		69.285 ± 0.756	77.962 ± 0.103	82.541 ± 0.364	85.628 ± 0.127
**M** **AL** **DB-CNN**		69.397 ± 0.695	77.233 ± 0.248	81.367 ± 0.230	83.873 ± 0.287
**MALDB**		69.523 ± 0.827	79.475 ± 0.519	85.067 ± 0.182	86.816 ± 0.174
